# Supplementary material for: Antiviral, Immunomodulatory and Antiproliferative Activities of Recombinant Soluble IFNAR2 without IFN-ß Mediation
Source: J Clin Med. 2020 Mar 31;9(4):959. doi: 10.3390/jcm9040959 (PMC7230527; doi:10.3390/jcm9040959)
Supplement: Supplementary file 1 [file jcm-09-00959-s001.pdf]

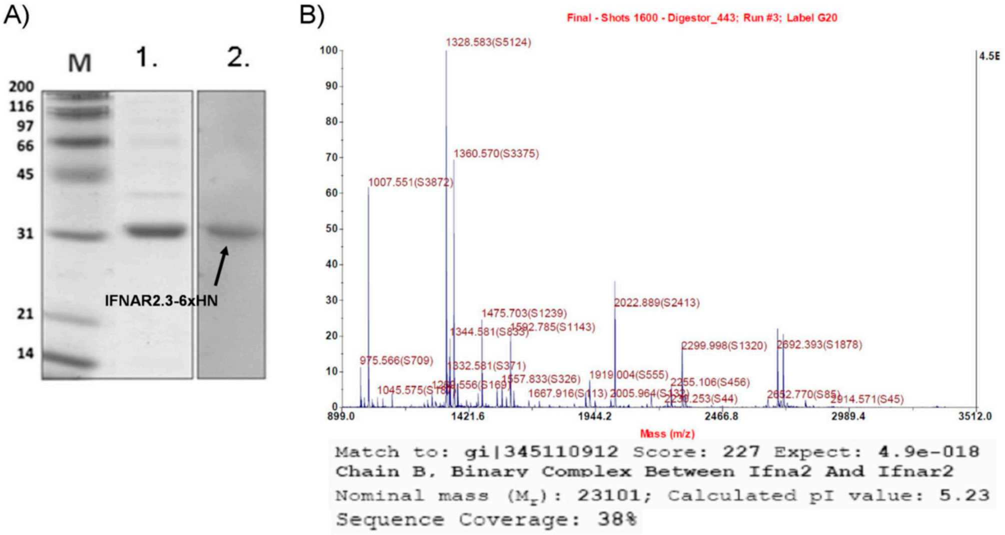

Supplementary Figure 1. (A) Recombinant sIFNAR2 purified. M: molecular weight, 1.SDS-PAGE 12%; 2. Western blot of purified sIFNAR2 (30 kDa). **(B)** Protein identification by MALDI-TOF MS and peptide mass fingerprinting. The Figure previously published in Suardíaz et al. (2016) recombinant soluble IFN receptor (sIFNAR2) exhibits intrinsic therapeutic efficacy in a murine model of Multiple Sclerosis. Neuropharmacology 110(Pt A):480–492.

Suplementary Table 1. Endotoxin levels of the recombinant sIFNAR2 at working dilution determined using the Endosafe®-PTS™ system and thekinetic chromogenic"Limulus Amebocyte Lysate" (LAL) Test Cartridges with a sensitivity of 0.005 EU mL^{-1}" (Charles River Laboratories). Endotoxin levels at the working dilutions used for all the experiments were less than 1 EU mL^{-1}, which is equivalent to a quantity of endotoxin lower than 0.1 ng mL^{-1}[20–22]".

Batch	Endotoxin at Woking Dilution (30µg mL^{-1})
Batch1	1.1 EU mL^{-1}
Bach 2	0.9 EU mL^{-1}
Bach 3	0.58 EU mL^{-1}
Bach 8	0.5 EU mL^{-1}

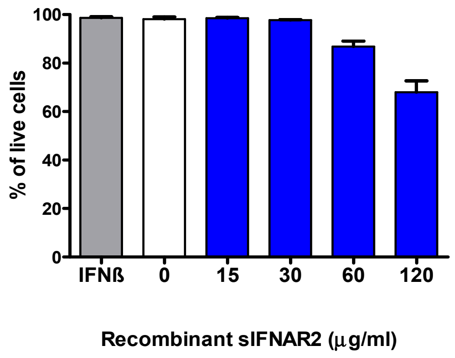

Supplementary Figure 2: Cell viability in the presence of recombinant sIFNAR2. PBMC were cultured in duplicate in the presence of different concentrations of recombinant sIFNAR2 (15, 30, 60 and 120µg mL^{-1}) during 24 h, using IFN-ß exposure (20UI mL^{-1}) as a positive control. Cells were stained with LIVE/DEAD® Fixable Red Far to select the living cells, following the manufacturer's instructions. The fixed cell suspension was analyzed by flow cytometry using the appropriate excitation and detection channel. The results are expressed as a percentage of live cells. There were

no differences in the percentage of live cells at 15, 30, nor 60 μg mL^{-1} of recombinant sIFNAR2 compared to IFN-ß exposure, and only a non-significant decrease was observed at 120 μg mL^{-1}, which is a concentration much higher than the working concentration used for all the experiments.

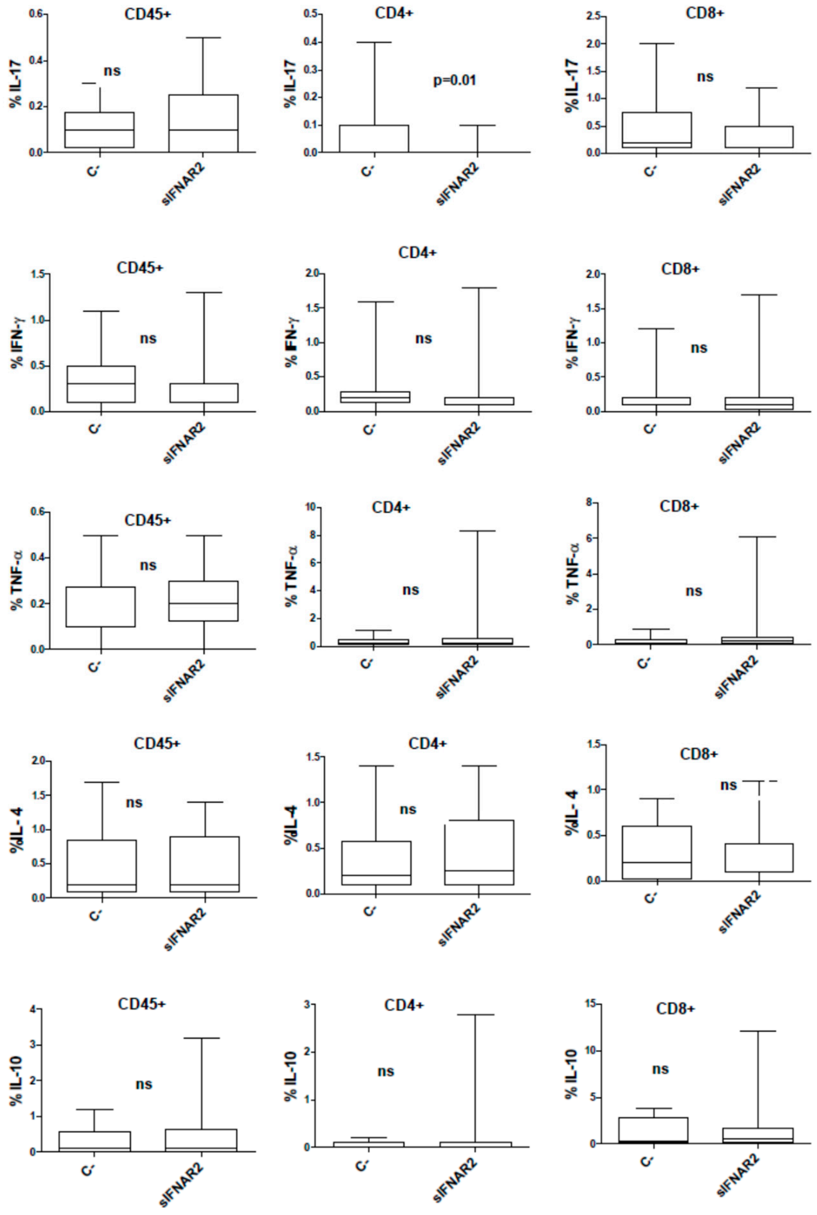

Supplementary Figure 3. Production of IL-17, IFN-γ, TNF-α, IL-4 and IL-10 in human PBMC in the presence of recombinant sIFNAR2. Intracellular cytokines in CD45+, CD4+ and CD8+ T cells were assessed by flow cytometry. The data show the percentage of positive cells expressing IL-17, IFN-γ, TNF-α, IL-4 or IL-10. The cells exposed to recombinant sIFNAR2 (at 30 μg mL^{-1}) were compared to cells without stimulation or negative control (c-) (Wilcoxon Rank test). N=10 * p< 0.01. ns: non-significant.

Supplementary RNA-seq data and analysis includes the differentially expressed genes (Fold change > 2) after sIFNAR2 addition used in a subsequent pathway enrichment analysis. The first 30 enriched pathways identified by IPA software are shown. Molecules, identified as putative regulators of this gene set, are also indicated together with its predicted activation state.
